# Comparison of learning outcomes of interprofessional education simulation with traditional single-profession education simulation: a mixed-methods study

**DOI:** 10.1186/s12909-022-03640-z

**Published:** 2022-08-30

**Authors:** Hui-Wen Chen, John M. O’Donnell, Yu-Jui Chiu, Yi-Chun Chen, Yi-No Kang, Yueh-Ting Tuan, Shu-Yu Kuo, Jen-Chieh Wu

**Affiliations:** 1 Department of Nursing, College of Nursing, National Yang Ming Chiao Tung University, Taipei, Taiwan; 2 Department of Nurse Anesthesia, University of Pittsburgh Nurse Anesthesia Program, Pittsburgh, Pennsylvania USA; 3 Winter Institute for Simulation, Education and Research (WISER) VB 360A, 230 McKee Place, Suite 300., PA 15213 Pittsburgh, USA; 4 Department of Emergency Medicine, Taipei Medical University Hospital, Taipei, Taiwan; 5 Department of Education and Humanities in Medicine, School of Medicine, College of Medicine, Taipei Medical University, Taipei, 110 Taiwan; 6 Institute of Health Policy and Management, College of Public Health, National Taiwan University, Taipei, Taiwan; 7 Department of Health Care Management, College of Health Technology, National Taipei University of Nursing Health Sciences, Taipei, Taiwan; 8 Department of Emergency Medicine, Taipei Medical University Hospital, Taipei, Taiwan; 9 School of Nursing, College of Nursing, Taipei Medical University, 250 Wuxing Street, Taipei, 11031 Taiwan; 10 Department of Nursing, Taipei Medical University Hospital Taipei Medical University, 252 Wuxing St., Taipei, 11031 Taiwan; 11 Department of Emergency, Taipei Medical University Hospital, 252 Wuxing Street, Taipei, 110301 Taiwan; 12 Department of Education and Humanities in Medicine, School of Medicine, College of Medicine, Taipei Medical University, 250 Wuxing Street, Taipei, 11031 Taiwan

**Keywords:** Interprofessional education, Medical student, Nursing student, Simulation, Curriculum development

## Abstract

**Background:**

Interprofessional collaborative practice is essential for meeting patients’ needs and improving their health outcomes; thus, the effectiveness of interprofessional education (IPE) should be clearly identified. There is insufficient evidence in the literature to determine the outcomes of IPE compared to traditional single-profession education (SPE). This study aimed to compare the outcomes of IPE and SPE during a simulation training course.

**Methods:**

The study design was a mixed-methods, incorporated cross-over design and a qualitative survey. A total of 54 students including 18 medical students and 36 nursing students were recruited from March to April 2019. The 4-week simulation course was designed based on Kolb’s experimental learning theory and Bandura’s social learning theory. Participants were evenly divided into group 1 (received IPE-learning followed by SPE-learning), and group 2 (received SPE-learning followed by IPE-learning). Students’ medical task performance, team behavior performance, teamwork attitude, and patient safety attitude were collected at pretest, mid-test, and posttest. Descriptive statistics and repeated measures analysis of variance were used. End-of-study qualitative feedback was collected, and content analysis was performed.

**Results:**

Both groups demonstrated moderate-to-large within-group improvements for multiple learning outcomes at mid-test. Group 1 students’ medical task performance (*F* = 97.25; *P* < 0.001) and team behavior performance (*F* = 31.17; *P* < 0.001) improved significantly. Group 2 students’ medical task performance (*F* = 77.77; *P* < 0.001), team behavior performance (*F* = 40.14; *P* < 0.001), and patient safety attitude (*F* = 6.82; *P* < 0.01) improved significantly. Outcome differences *between groups* were nonsignificant. Qualitative themes identified included: personal factor, professional factor, interprofessional relationship, and learning. The IPE program provided students with exposure to other professions and revealed differences in expertise and responsibilities.

**Conclusion:**

IPE-simulation and SPE-simulation were effective interventions that enabled medical and nursing students to develop critical medical management and team behavior performance. IPE-simulation provided more opportunities for improving competencies in interprofessional collaborative practice. In circumstances with limited teaching resources, SPE-simulation can be an acceptable alternative to IPE-simulation.

**Supplementary Information:**

The online version contains supplementary material available at 10.1186/s12909-022-03640-z.

## Introduction

Interprofessional collaborative practice involving multiple professionals is essential to the provision of substantive care that responds to patients' needs and improves their health outcomes [1]. The World Health Organization and other policymakers worldwide have advocated for the essential role of interprofessional education (IPE) in transforming health professionals into a “collaborative practice-ready” health workforce; such practice enhances health systems and enables local health needs to be met [2]. The development of standardized training methods for effective IPE training for health care professionals should be a key objective. Studies worldwide have proposed practice models for IPE delivery; however, these models have achieved varying outcomes [3]. Further investigation should be conducted to identify more effective methods for providing standardized and effective IPE.

IPE involves members of two or more professions learning together and from each other, such that effective collaboration and improved health outcomes can be achieved [2]. The most common methods for implementing IPE include classroom-based lectures, case-based discussions, community-based experience sharing, role-playing and simulation [4]. IPE is thought to be an effective strategy in part because it provides opportunities for learners to learn from each other’s behavior [5, 6], learn from their experiences [7], increases their motivation [6, 8] and allows interaction with different disciplines [9] with these key elements supported by social learning theory [5, 6], experiential learning theory [7], adult learning theory [6, 8], and social construct theories [9] respectively. Simulation methods in deploying IPE education is essential because it allows for knowledge to be imparted and retained more effectively [10].

The existing body of evidence suggests that IPE simulation is an effective educational methodology [3, 11]. However, most studies have applied single-group pretest and posttest designs. Although some studies have compared the learning outcomes of IPE simulation with those of other IPE teaching methods, few studies have compared the outcomes of IPE and traditional non-IPE methods that involve only one profession. Further, the relative influence of observing, interacting, reflecting, and discussing with different disciplines during IPE learning experiences has not been clearly delineated in the literature.

The present study compared an IPE simulation model involving multiple professions with simulation involving only a single profession to determine whether the IPE simulation model produces more effective learning outcomes in terms of medical task performance, team behavior performance, teamwork attitude, and patient safety attitude among medical and nursing students.

## Methods

### Theoretical framework

This study is based on Bandura’s social learning theory and Kolb’s experiential learning theory. New behaviors can be acquired through direct experience, by observing others behavior within a social context, or the observation of the consequences of the behavior by others [5]. There are four important processes which contribute to successful learning, including attention, retention, reproduction, and motivation. In Kolb’s experiential learning theory, learners’ knowledge is created by the process of experience transformation, consisting of four key stages: concrete experience, reflective observation, abstract conceptualization, and active experimentation [7, 12]. Accordingly, we designed and implemented a simulation-based IPE training program in our study to provide learning opportunities for students from different disciplines to learn from each other, become actively engaged in the simulation activities, and to observe each other’s behavior. Further, each student was provided the opportunity to reflect on their own performance in the simulated scenario and plan for improvement during both faculty-guided and self-directed debriefing sessions followed by repetitive practice in order to develop desirable collaborative behavior.

### Study design and participants

We adopted a mixed-methods design, incorporated cross-over design and a qualitative survey to evaluate medical and nursing students who participated in a 4-week simulation course at a medical university in Northern Taiwan in 2019. This study design is a reasonable approach as physicians and nurses together represent the two largest groups of healthcare providers in the world, and a majority of IPE activities described in the literature incorporates medical and nursing professions or students as participants [4]. The grounded theory approach was used for the qualitative survey on participants’ learning process throughout the course. The study was approved by the institutional review board of the conducting institution (IRB #N201610015). Informed consent was obtained from all students after the explanation of the purposes and procedure of the study.

The study participants comprised 18 medical and 36 nursing students. The medical students were in their fifth year of a 6-year medical school curriculum, and they were taking part in a clerkship rotation at either the emergency or internal medicine departments of a teaching hospital. The nursing students were in the last semester of their 4-year Bachelor of Science in Nursing (BSN) program. All the medical students and nursing students were certified as advanced cardiac life support and basic life support providers by the Taiwan Society of Emergency & Critical Care Medicine.

**Fig. 1:**
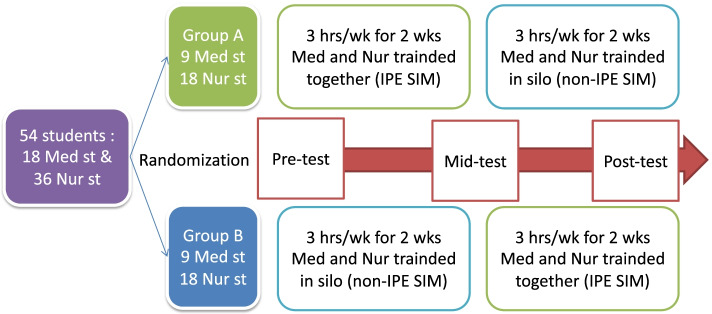
Interprofessional education (IPE) and Single-profession education (SPE) sessions. Note: *Med st* Medical students, *Nur st* Nursing students, *IPE SIM* Interprofessional education Simulation, *SPE SIM* Single-profession education Simulation

We developed a structured 4-week simulation course (3 h per week) that incorporated a 2-week IPE program during which medical and nursing students were trained together about how to manage a critical patient in a ward scenario as first responders as well as a 2-week single-profession education (SPE) program during which medical and nursing students were trained separately (Fig. 1). The course instruction was team-based and included both nursing and medical personnel. Each medical student was assigned a number from 1 to 18, and each nursing student was assigned a number from 1 to 36. All students were then randomly and evenly divided using Microsoft Excel’s random number generator (Microsoft, Seattle, WA, USA) into 3-member teams so that each team comprised one medical and two nursing students. The first 27 students (Group 1, 9 teams) underwent a 2-week (6-h) IPE program followed by a 2-week (6-h) SPE program. The remaining students (Group 2, 9 teams) underwent the 2-week (6-h) SPE program followed by the 2-week (6-h) IPE program (Fig. 1).

### Simulation course development

The topics covered within the course comprised the core competencies for the initial assessment of critical patients including primary airway management, patient reevaluation, initial cardiac arrest management, and teamwork concepts based on the American Heart Association (AHA) Guidelines for cardiopulmonary resuscitation and emergency cardiac care. Further, principles of the TeamSTEPPS (Team Strategies and Tools to Enhance Performance and Patient Safety) 2.0 program established by the United States Agency for Healthcare Research and Quality [13, 14] were incorporated. To maintain training consistency, all faculty members received training to provide step-by-step scripted guidance. The simulation course design was reviewed by an expert panel, which consisted of physicians, nurse practitioners, registered nurses, and a physician internationally certified as a Certified Healthcare Simulation Educator or CHSE (Society for Simulation in Healthcare Certification Department). All clinical experts had at least 10 years of experience working in emergency departments and 5 years of simulation teaching experience.

The 4-week simulation training course was constructed using a scaffolding model so that the students could develop their self-efficacy and competencies. The process was structured to present a range of difficulty from easy to complex. The participants had multiple opportunities to manage the simulated patient as a medical team in order to practice and retain their learned skills and behaviors effectively. At the beginning of the simulation-based training program, we engaged and motivated the students by briefing the importance of the training objectives and pointed out their connection to actual clinical responsibilities using video exemplars and group discussion. In each scenario, some students were assigned to manage the simulated patient while the rest of the students were assigned to be observers. These students could observe participating students’ behaviors and the consequences of their performance. In each simulation round, the students experiencing the simulated scenario (concrete experience), had discussion within learning group in a structured faculty-lead debriefing session using the GAS (gather, analyze, and summarize) model approach [15] with reflective observation and abstract conceptualization incorporated into the debriefings. They then were able to practice the simulated scenario again (active experimentation) with the design elements based on Kolb’s experiential learning cycle.

### Assessment instruments and data collection

The primary learning outcomes included medical task performance (MTP), team behavior performance (TBP), teamwork attitude (TA), patient safety attitude (PSA) related to teamwork and communication, and qualitative feedback related to the course experience. The expert panel designed, adopted, or modified assessment instruments according to the students’ learning level and specific training objectives in alignment with the International Nursing Association of Clinical Simulation and Learning (INACSL) Standards for Best Practice™ [16, 17]. Specifically, an expert panel consisting of two clinical resuscitation experts and two team training experts established the content validity of the assessment tools through a consensus-setting process. All outcomes were assessed at pretest, mid-test (one week after completion of the first 2-week program), and posttest (one week after completion of the 4-week course). Two trained faculty raters evaluated team performance during a standardized 15-min simulation scenario. For pretest rater calibration, the two raters scored a standardized videotaped simulation scenario which had been expertly scored. They then met to review their scores, compare their results with the expert scoring and to identify key challenge points in rating in order to standardize their scoring of observed behaviors. Interrater reliability overall between the two raters was 0.83 indicating a high level of agreement.

#### Medical task performance (MTP)

Our expert panel modified a standardized 54-item checklist to assess the students’ MTP. This checklist was based on the Taiwanese version of the AHA’s resuscitation guidelines. The domains of the checklist consisted of initial assessment, care environment setting evaluation, airway management, patient reevaluation, and cardiac arrest management. The checklist was rated on a dichotomous scale with 1(Yes) and 0 (No), and total score was between 0 and 54.

#### Team behavior performance (TBP)

A 10-item rating scale based on a modified TeamSTEPPS Performance Observation Tool was adapted for local conditions and with respect to cultural differences. Faculty raters used the 10-item tool to score the students’ TBP on a 5-point scale (from 1 for very poor to 5 for excellent), with the total score ranging from 10 to 50. The rating scale consisted of two items for each of the domains, namely team structure, leadership, mutual support, situation monitoring, and communication.

#### Teamwork attitude (TA)

TA was measured using a modified 26-item version of the Teamwork Attitudes Questionnaire [18]. We deleted five items from the original 31-item version to be appropriate for our training program and local culture. The students rated their perception of teamwork attitudes on a 5-point scale from 1 (strongly disagree) to 5 (strongly agree). The Cronbach’s alpha of this questionnaire was calculated as 0.872.

#### Patient safety attitude (PSA)

A 12-item self-reported questionnaire was adopted based on the AHRQ’s (Agency for Healthcare Research and Quality) Hospital Survey on Patient Safety [19]. The study selected items from the original questionnaire that were suitable for medical and nursing students, namely those related to communication within care teams and teamwork. PSA was assessed on a 5-point scale from 1 (strongly disagree) to 5 (strongly agree). The Cronbach's alpha of this questionnaire was calculated as 0.680.

#### Qualitative data collection- reflection on the IPE course

To evaluate how the participants acquired a broader understanding of IPE principles, we adopted the grounded theory approach and collected trainees’ learning reflections through open-ended questions after they completed the course. The open-ended questions were useful to explore and obtain what students felt and kept in their mind, and researchers could map and verify what students may learn from the course by the responses [20]. Literature showed that open-ended questions with anonymous design could help to acquire more reliable responses [20]. An individual questionnaire was thus sent to each student, instead of conducting a face-to-face interview to reduce social expectation bias and hierarchical pressure among study participants. In order to avoid selection bias, every student was invited (using open-response items) to write down their reflections regarding knowledge acquisition and overall perceptions of the learning experience. These open-response items focused on revealing their experiences with respect to learning and working with members of the other professions as well as those from their own profession. All subjects returned the questionnaire and provided their reflections. It is important to have multiple questions for a clearer understanding and more reliable findings in a qualitative study [21]. In this study, we also used verification strategies such as an adequate data collection method and an appropriate sample [22]. Specifically, the open-ended questions used in this study were as follows: 1. Overall, what did I learn from the course? 2. Please share the learning experiences, advantages, and disadvantages with students from different disciplines (medicine or nursing) in this course? 3. Please share the experiences of learning medical management with students from different disciplines (medicine or nursing) in this course? 4. Please share the experiences of learning teamwork with students from different disciplines (medicine or nursing) in this course?

### Data analysis

Categorical variables are reported as frequency and percentage. Continuous variables are reported as means and standard deviations. A chi-squared test or independent-samples t-test was used to examine between-group differences at pretest. Because the data were collected at three time points, repeated-measures analysis of variance (RM-ANOVA) was used to explore *within-gro*up changes, and two-way RM-ANOVA was used to examine the corresponding *between-group* changes. To avoid type I error, Bonferroni adjustment was performed for multiple pairwise comparisons through RM-ANOVAs. In addition, partial η^2^ was calculated for the effect size. The data analyses of the present study were performed using SPSS version 22 (IBM, Armonk, NY, USA). Statistical significance was established a priori as a *P* value of < 0.05.

Qualitative data were analyzed using content analysis method by two independent coders (authors YK and SK). They first extracted meaningful elements from each sentence independently. Subsequently, they met and determined the formal definitions pertaining to the terminology for the categories of the elements. Students' responses were then coded using categories for IPE at the personal, professional, and interprofessional levels [23]. The coders also identified the new categories and themes within the data. The qualitative data was subsequently grouped into themes and subthemes based on the codes. A consensus regarding the themes and their respective definitions was reached through in-depth discussions. All the authors reviewed and verified the posttest categories of the themes and their responses.

## Results

In total, 54 students completed all tests and training protocols in the present study. No significant between-group differences were observed for sex or student type. The mean scores for medical task performance, team behavior performance, teamwork attitude, and patient safety attitude were similar between the two groups at pretest (Table 1). Notably, at pretest, most students recognized the essential role of patient safety attitude and had a positive attitude toward teamwork in clinical practice (Table 1).

**Table 1:** Demographic characteristics and pretest scores of the two groups (*n* = 54)

Variable	Group 1	Group 2	*p*
**Sex**			> .05
Male, n (%)	8 (30)	8 (30)	
Female, n (%)	19 (70)	19 (70)	
**Student Type**			> .05
Medical student, n (%)	9 (33)	9 (33)	
Nursing student, n (%)	18 (67)	18 (67)	
**Pretest score (mean ± SD)**			
Medical task performance	18.78 ± 6.16	20.44 ± 3.58	> .05
Team behavior	1.97 ± 0.36	1.87 ± 0.45	> .05
Team attitude	4.23 ± 0.32	4.20 ± 0.35	> .05
Patient safety	3.82 ± 0.31	3.74 ± 0.45	> .05

**Note:** Group 1 underwent IPE simulation followed by SPE simulation

Group 2 underwent SPE simulation followed by IPE simulation

*IPE* Interprofessional education, *SPE* Single-profession education, *SD* Standard deviation

**Fig. 2:**
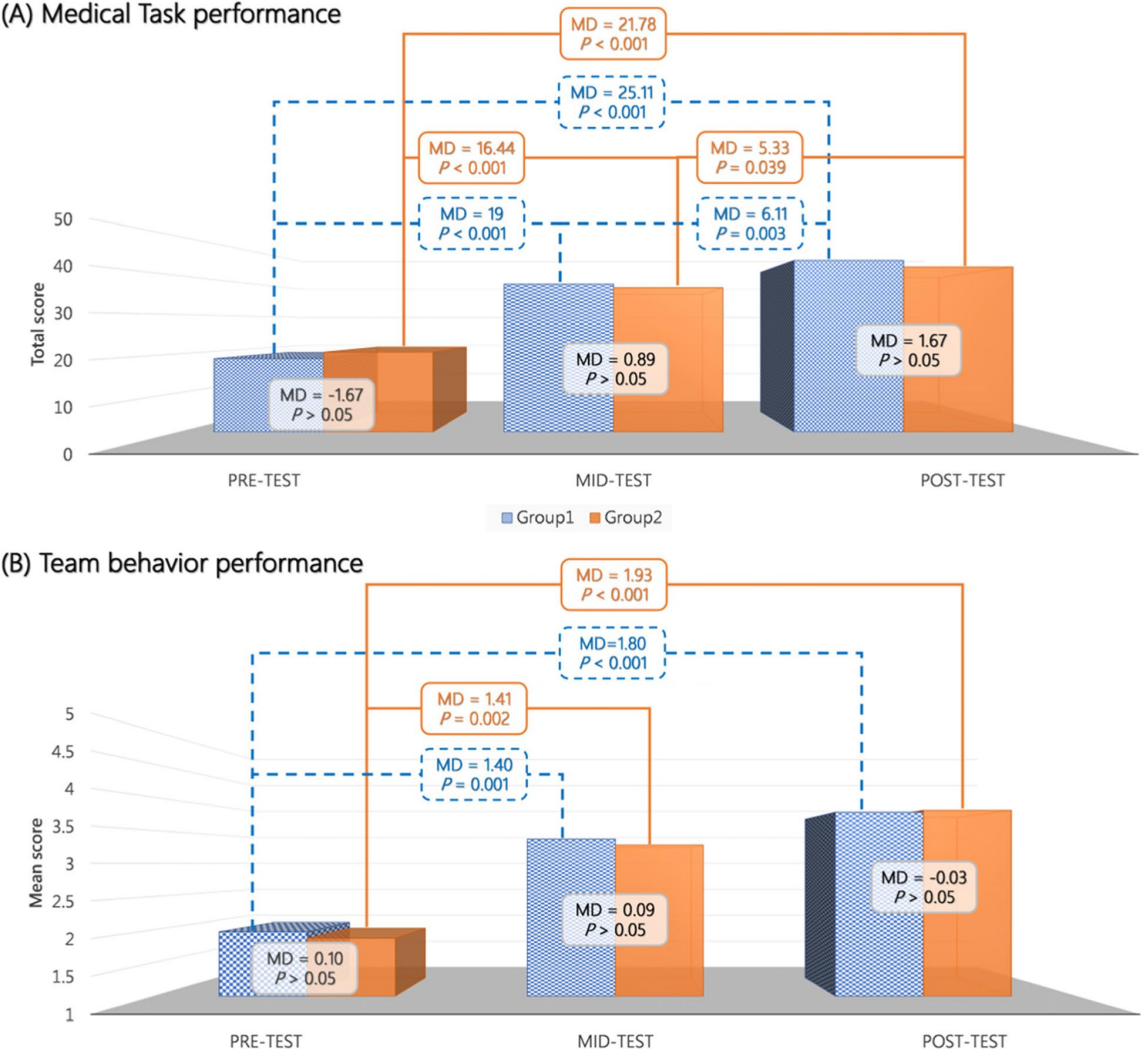
Repeated measures analysis of variance for (**A**) medical task performance and (**B**) team behavior performance. Blue bars indicate results for Group 1 (received IPE simulation followed by SPE simulation); orange bars indicate results for Group 2 (received SPE simulation followed by IPE simulation). MD, mean difference; IPE, interprofessional education; SPE, single-profession education

**Fig. 3:**
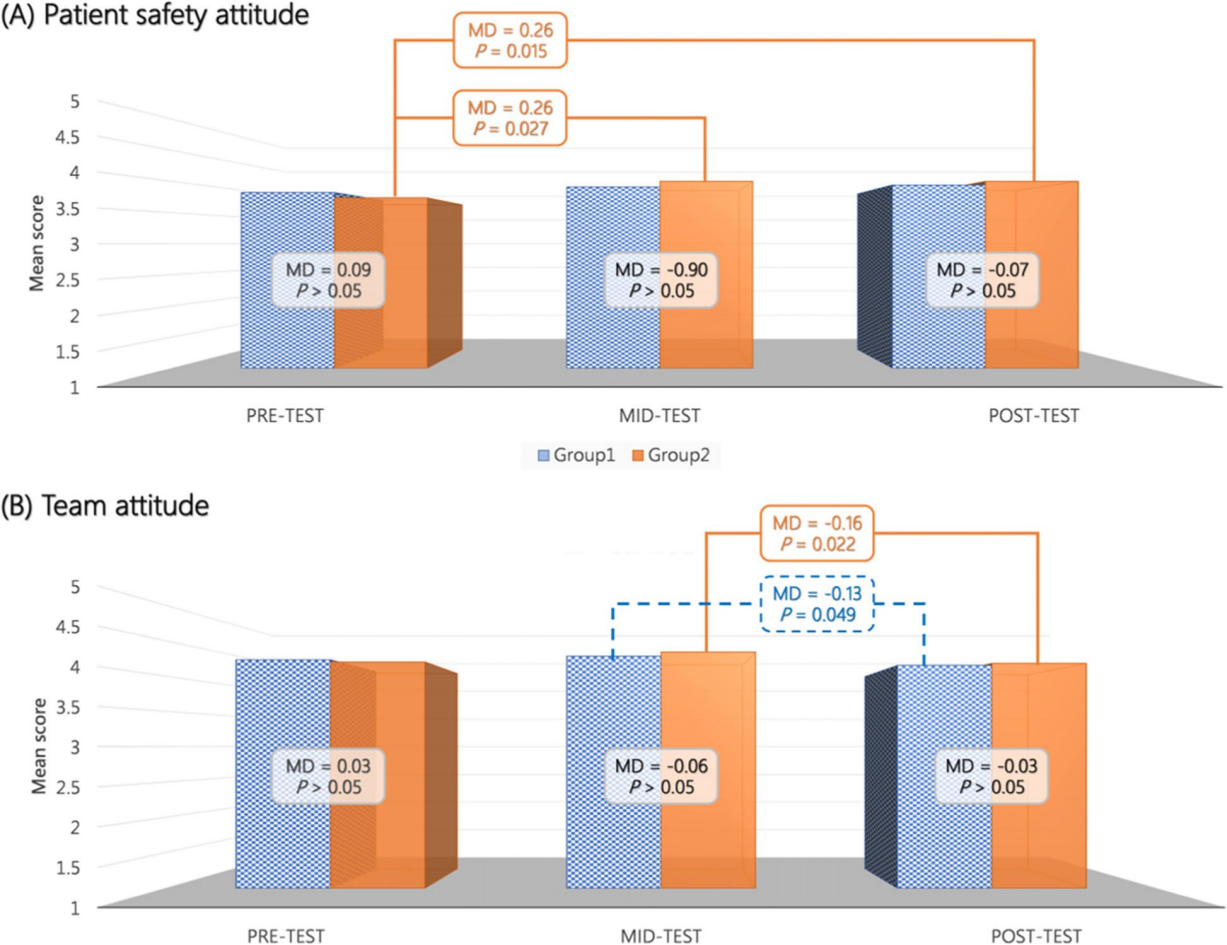
Repeated measures analysis of variance for (**A**) patient safety attitude and (**B**) team attitude. Blue bars indicate results for Group 1 (received IPE simulation followed by SPE simulation); orange bars indicate results for Group 2 (received SPE simulation followed by IPE simulation). MD, mean difference; IPE, interprofessional education; SPE, single-profession education

The within-group comparison indicated that the students in both groups showed improvement in multiple areas after undergoing the simulation course (Figs. 2 and 3). In Group 1 (students who received IPE followed by SPE), significant improvements were observed for MTP (*F* = 97.25; *P* < 0.001; partial η^2^ = 0.92) and TBP (*F* = 31.17; *P* < 0.001; partial η^2^ = 0.80), but not for teamwork attitude and PSA. Although overall RM-ANOVA did not reveal any significant changes in teamwork attitude, post hoc analysis indicated that the posttest scores were higher than the mid-test scores (Mean Difference, MD =  − 0.13; *P* < 0.05). For the MTP of Group 1, posttest scores were significantly higher than pretest (MD = 25.11; *P* < 0.001) and mid-test (MD = 6.11; *P* < 0.01) scores. Moreover, for MTP, the mid-test score was significantly higher than the pretest score (MD = 19.00; *P* < 0.001). Significant changes were observed in TBP, with the posttest (MD = 1.80; *P* < 0.001) and mid-test (MD = 1.40; *P* < 0.001) scores being significantly higher than the pretest scores. However, no significant differences were noted in TBP between posttest and mid-test (MD = 0.40; *P* > 0.05).

In Group 2 (students who received SPE followed by IPE), significant improvements in MTP (*F* = 77.77; *P* < 0.001; partial η^2^ = 0.95), TBP (*F* = 40.14; *P* < 0.001; partial η^2^ = 0.93), and PSA (*F* = 6.82; *P* < 0.01; partial η^2^ = 0.28) were observed. Interestingly, a significant decrease in teamwork attitude (posttest) was also observed (*F* = 4.10; *P* < 0.05; partial η^2^ = 0.27). Specifically, the score for MTP at posttest was significantly higher than the scores at pretest (MD = 21.78; *P* < 0.001) and mid-test (MD = 5.33; *P* < 0.05). The score for MTP at mid-test was also significantly higher than the score at pretest (MD = 16.44; *P* < 0.001). In Group 2, the students scored higher for TBP at mid-test (MD = 1.93; *P* < 0.001) and posttest (MD = 1.41; *P* < 0.01) relative to their pretest scores; however, no significant difference was observed in TBP (MD = 0.52; *P* > 0.05) between the mid-test and posttest time points. Similarly, the mid-test (MD = 0.26; *P* < 0.05) and posttest (MD = 0.26; *P* < 0.05) scores for PSA were significantly higher than the pretest score, but no significant difference was detected between the mid-test and posttest scores (MD = 0.003; *P* > 0.05). The students in Group 2 scored lower for teamwork attitude at posttest than at mid-test (MD =  − 0.16; *P* < 0.05), and no significant differences were observed between posttest and pretest scores and between mid-test and pretest scores.

The mid-test and posttest results for both groups indicated moderate-to-high scores for MTP, TBP, TA, and PSA. The between-group differences in these outcomes were nonsignificant (Table 2). There were also nonsignificant differences in in MTP (*F* = 0.05; mean difference [MD] = 0.30; *P* > 0.05), TBP (*F* = 0.16; MD = 3.03; *P* > 0.05), TA (*F* = 0.06; MD =  − 0.02; *P* > 0.05), and PSA (*F* = 0.09; MD =  − 0.02; *P* > 0.05) between the IPE and SPE groups. For profession-specific results of PSA and TA, please refer to supplementary figure S1 and S2.

**Table 2:** Descriptive statistics and independent-samples *t*-test of mid-test and posttest scores of Groups 1 and 2

Outcome	Mid-test			Post-test		
(mean ± SD)	Group 1	Group 2	*p*	Group 1	Group 2	*p*
Medical task performance	37.78 ± 2.64	36.89 ± 5.49	*P* > 0.05	43.89 ± 2.71	42.22 ± 4.12	*P* > 0.05
Team behavior	3.37 ± 0.53	3.28 ± 0.44	*P* > 0.05	3.77 ± 0.58	3.80 ± 0.53	*P* > 0.05
Team attitude	4.28 ± 0.30	4.34 ± 0.32	*P* > 0.05	4.15 ± 0.35	4.18 ± 0.37	*P* > 0.05
Patient safety	3.91 ± 0.31	4.00 ± 0.40	*P* > 0.05	3.94 ± 0.35	4.00 ± 0.32	*P* > 0.05

**Note:** Group 1 underwent IPE simulation followed by SPE simulation

Group 2 underwent SPE simulation followed by IPE simulation

*IPE* Interprofessional education, *SPE* Single-profession education, *SD* Standard deviation

### Qualitative reflection on IPE course

Four major themes were identified with respect to the learning experiences derived from this IPE simulation course, namely personal factor, professional factor, interprofessional relationship, and learning (Table 3). The benefits of IPE simulation were identified from the open-ended responses. The most frequently reported benefit was that this course provided a good opportunity for the students to understand and learn about the expertise and responsibilities of each other’s profession.

**Table 3:** Learning experiences from an interprofessional education (IPE) simulation course for medical and nursing students

Themes	Subthemes	Quotes
1. Personal factors	Role	- “The physician and nurse play different roles to manage the first five-minute situations.”
		- “After taking these 6 courses, I realized the role of being a leader.”
	Responsibility	- “I know better about the job content of nurse than before I took this course.”
		- “I know to complete the physical assessment and initial management before calling for help from senior.”
		- “I know my responsibility during resuscitation after this class.”
	Emotion	- “I realized that I am afraid of communicating with others.”
		- “After class, I have more empathy toward each other.”
		- “I felt panic when being asked questions initially.”
		- “I felt respected.”
		- “It is important to remain calm and thinking logically.”
	Limitation	- “I can’t focus fully on owns task.”
		- “It takes time to be familiar with each other.”
		- “It was difficult in communication with other disciplinary.”
	Assumptions	- “I thought there always some barriers between physician and nurses.”
		- “I am not comfortable to be a leader. After receiving support in my class, I felt better.”
		- “Leader should respect his teammate.”
2. Professional factors	Identity	- “I notice there exist different thinking process in physician and nurse.”
		- “Medical student usually has a clam and professional attitude.”
		- “Nursing student is good at IV, procedure preparation, taking vital sign.”
	Role	- “Nursing student approaches patient in detail.”
		- “It is important to be a leader, provide feedback and remind others”
	Importance of his/her profession	- “Nursing students know the patient’s condition very well and they are alert.”
		- “I realize the strength and limitation of other disciplinary.”
		- “knowing the differences and importance of nurse/physician job
3.Interprofessional relationship	Role	- Nursing students tend to act faster and are more quick-witted than medical students
		- Medical students are better leaders than nursing students
		- We get to understand each other better through the class
	Teamwork	- Teamwork is essential
		- I learned how to work with our teammates
		- It is crucial that everyone works together toward the same goal
		- I know how we can assist each other
	Communication	- We can communicate as equals for the good of the patient instead of simply following orders or accepting a top-down system
		- Effective communication between team members enables efficient teamwork
		- Feedback between teammates is crucial
		- At the start the class, we could not speak up; but by the end of this course, we could communicate effectively
	Patient care	- We work together to improve the patient’s condition
		- In a critical situation, good coordination between physicians and nurses enables better medical management for a patient
4. Learning	Opportunity of learning with/from other	- In this course, I learned to see things from the other profession’s perspective
		- This is my first time learning with members of another profession
		- I have a chance to learn how to interact with members of other disciplines
	Real situation	- This course simulates clinical scenarios
		- The clinical interdisciplinary scenario mimics a hospital work environment
		- I wish to have more interactions with others (members of other professions)
		- The realistic, repeated practices were helpful

## Discussion

This mixed-methods study verified the effects of IPE simulation training designed to improve participants’ MTP and TBP, but not team work attitude. Its findings demonstrated that both IPE and SPE simulations were effective in enhancing medical and nursing students’ medical task performance (MTP) and teamwork behavior performance (TBP); however, no significant between-group differences were observed in these two parameters. The strengths of this novel study lie in the development and design of this IPE program targeting medical and nursing students to learn about and from each other in multiple personal, professional, and interprofessional aspects.

The findings revealed that IPE simulation effectively improved TBP and verified the finding of interventional studies regarding the effectiveness of IPE courses in improving team performance. However, previous studies have mostly used single-group pretest–posttest designs [24]. Moreover, direct comparison of multiple-profession and single-profession training models is rare [3]. A 2020 systematic review [24] of 17 IPE training studies that used single-arm pretest–posttest designs reported that IPE is an effective intervention [25–38]; however, none of these studies compared the learning outcomes of IPE with SPE models for multiple activities or directly compared the outcomes of IPE and SPE simulations. A study comparing interprofessional and uniprofessional education related to resuscitation skills reported no significant difference between the interprofessional group and the uniprofessional group [39]; however, its objective performance assessment was only conducted at posttest, and no pretest–posttest comparisons were performed. Therefore, in our study, objective assessments were conducted to obtain more comprehensive findings regarding the differences between IPE and SPE simulation training, thus contributing to the literature. In improving medical task performance and team behavior performance, simulation-based SPE was equally effective to simulation-based IPE among medical and nursing students.

A study in the United Kingdom reported no difference between IPE and uniprofessional learning with respect to team dynamics and resuscitation tasks but students learned about multiple aspects of interprofessional collaboration competency [39]. Nevertheless, students’ learning styles and skills and faculties’ teaching models and skills can vary among different cultures [40], and these factors may affect learning outcomes [41]. Our study produced similar findings to the aforementioned studies including personal, professional, and interprofessional aspects of collaborative practice, suggesting that the learning effects of IPE are similar in both Western and Eastern cultures. Simulation-based IPE can be used to develop the interprofessional competency of medical and nursing students.

Simulation-based IPE provides opportunities for students from different disciplines to interact with each other, observe others’ behaviors and understand each other’s mental processes through discussion and reflection. However, this does not seem to impact on the development of medical and nursing students’ medical task performance and team behavior significantly when compared to simulation-based SPE. Simulation education provides substantially improved outcomes for task and team behavior training relative to other training methods [42], which may explain why multiple-profession learning did not significantly contribute to training outcomes between groups in the present study. Furthermore, the participating students were also undergraduates with limited clinical experience; therefore, positive learning outcomes may have been achieved with any effective intervention method due to the large potential effect size. This provides faculty with the knowledge that single profession simulation training can be as effective as interprofessional simulation training if targeting outcomes are medical task performance and team behavior. This finding may offer faculty more flexibility in developing curriculum. Including more students from different professions to train together requires more coordination, is more costly from a time and space perspective [4, 43], and may be more challenging due to the restriction of social distancing during the ongoing pandemic [44]. Sequencing medical task performance competency and team behavior skill acquisition within their own profession and then initiating interprofessional activity to actually practice their skills with other professions in team-based learning to enhance multiple aspects of interprofessional collaboration competency would be a reasonable option.

The present study also revealed that medical and nursing students’ TBP significantly improved after the intervention, but no significant improvements were noted for TA and PSA related to teamwork and communication in either the IPE or SPE groups. The use of IPE could improve attitudes toward teamwork and collaboration among various health care professionals [45]. Simulation-based IPE is also recognized as an effective model for improving practicing physicians and nurses’ teamwork attitudes [37, 38]. However, our finding may be limited by a ceiling effect because our students reported a high score of TA and PSA at pretest. Coster et al. discovered that interprofessional attitude declined over time in students from multiple disciplines, and that the reinforcement of this attitude may be related to their attitude toward IPE at pretest [46]. Moreover, students might have received additional exposure and experiences during their clinical rotations, and such experiences could have influenced attitudes toward teamwork and patient safety, which is a form of maturation bias. Further studies focus on potential reasons underlying students’ teamwork attitude and patient safety attitude are warranted.

On the other hand, our qualitative results indicated that our students improved in terms of MTP, team behavior, and other interprofessional components. The qualitative results echo the findings in previous studies, in which simulation training appears to improve interprofessional work, clinical skills, reflective practice, leadership, teamwork, better understanding of each other’s roles, and communication skills [30, 47]. In the present study, students reported that this IPE experience helped to overcome the communication barrier between physicians and nurses, helped them develop more empathy for each other’s profession, and established shared mental models for the two professions. A recent study examined a model for developing interprofessional empathy and discovered that interprofessional empathy influences the performance of interprofessional teams [48]. Our interprofessional learning activity allowed medical and nursing students to interact with each other and observe each other’s behaviors and their consequences in a safe and controlled environment. During faculty-facilitated debriefing sessions, these medical and nursing students could share their feelings, thoughts, and suggestions for improving professional performance and teamwork in a psychologically safe atmosphere. They could also modify and apply their teamwork behaviors by repeating practice scenarios. Our results indicated that a simulation-based IPE curriculum designed and grounded around Bandura’s social learning theory and Kolb’s experiential learning theory is an effective learning method that enables students to develop interprofessional competency; however, IPE activities require more resources relative to traditional single-profession learning activities.

## Limitations

Our study has several limitations that are relevant to the interpretation of our results. First, it involved a limited number of participants and small-sized teams with a specific proportion of team members (one medical student and two nursing students as a team). Second, during the study period, the students were undergoing various clinical rotations, which could have affected the test outcomes. Furthermore, no follow-up assessment of the participating students was conducted to evaluate changes in their learning and attitude after the completion of the course. Future studies examining long-term learning outcomes should be conducted.

## Conclusions

Interprofessional education simulation was an effective intervention for teams of medical and nursing students to improve their medical task performance and team behavior performance and to develop interprofessional collaborative practice competencies. When resources are limited, single-profession education would be an acceptable alternative pathway for training of medical task performance and team behavior performance.

## Supplementary Information

**Additional file 1:** **Supplementary Figure S1.** Repeated measures analysis of variance of patient safety attitude for medical students and nursing students. Blue bars indicate results for Group 1 (received IPE simulation followed by SPE simulation); orange bars indicate results for Group 2 (received SPE simulation followed by IPE simulation). MD, mean difference; IPE, interprofessional education; SPE, single-profession education.

**Additional file 2:** **Supplementary Figure S2.** Repeated measures analysis of variance of team attitude for medical students and nursing students. Blue bars indicate results for Group 1 (received IPE simulation followed by SPE simulation); orange bars indicate results for Group 2 (received SPE simulation followed by IPE simulation). MD, mean difference; IPE, interprofessional education; SPE, single-profession education.

## Data Availability

All data generated or analyzed during this study are included in this published article.

## Abbreviations

IPE: Interprofessional Education
SPE: Single-Profession Education
TeamSTEPPS: Team Strategies and Tools to Enhance Performance and Patient Safety
GAS: Gather, Analyze, and Summarize
MTP: Medical task performance
TBP: Team behavior performance
TA: Teamwork attitude
PSA: Patient safety attitude
AHRQ: Agency for Healthcare Research and Quality
RM-ANOVA: Repeated-measures analysis of variance
MD: Mean Difference
