# Supplementary material for: Comparison of learning outcomes of interprofessional education simulation with traditional single-profession education simulation: a mixed-methods study
Source: BMC Med Educ. 2022 Aug 30;22:651. doi: 10.1186/s12909-022-03640-z (PMC9429663; doi:10.1186/s12909-022-03640-z)
Supplement: Supplementary file 2 — Additional file 2: Supplementary Figure S2. Repeated measures analysis of variance of team attitude for medical students and nursing students. Blue bars indicate results for Group 1 (received IPE simulation followed by SPE simulation); orange bars indicate results for Group 2 (received SPE simulation followed by IPE simulation). MD, mean difference; IPE, interprofessional education; SPE, single-profession education. [file 12909_2022_3640_MOESM2_ESM.docx]

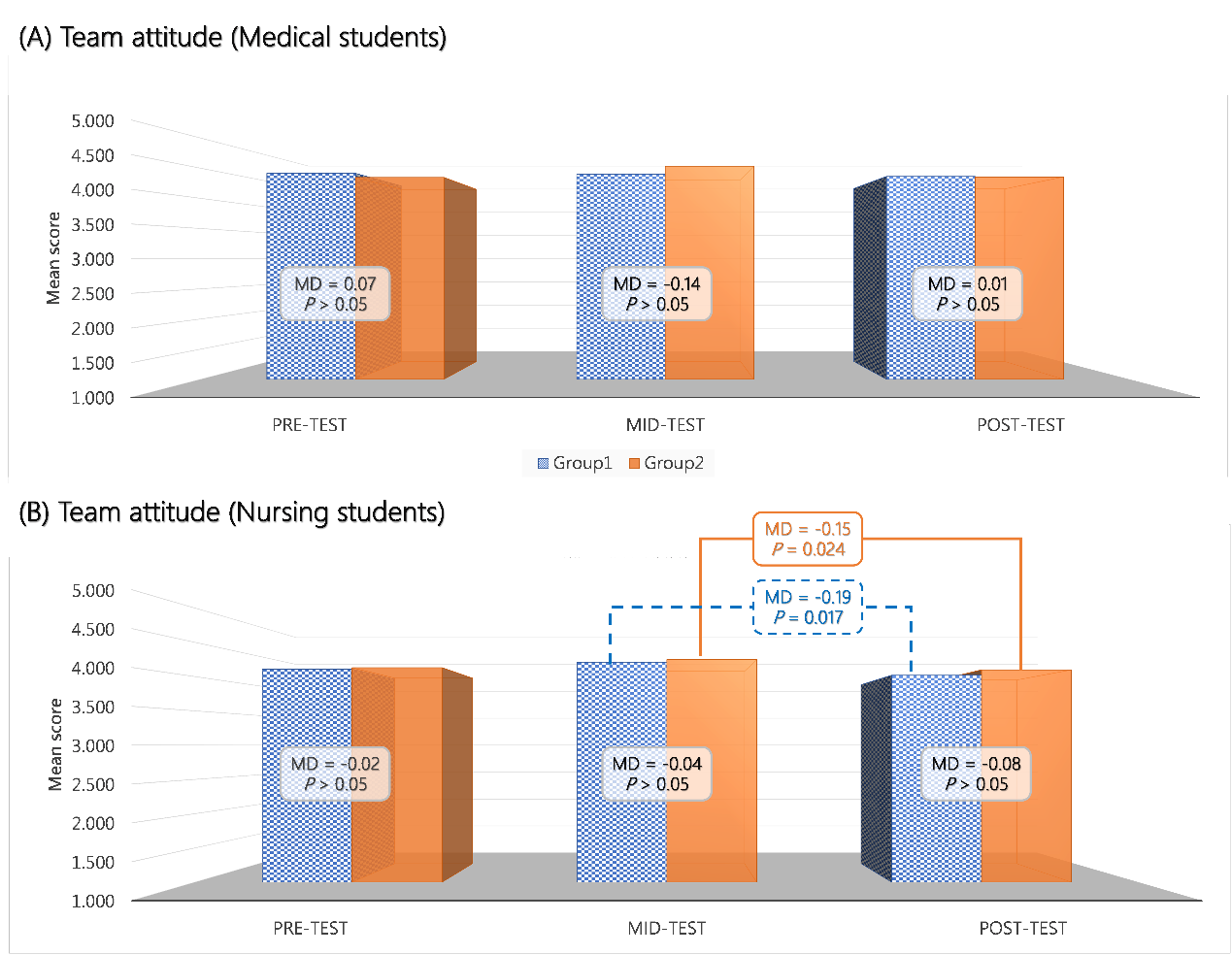


**Supplementary Figure S2.** Repeated measures analysis of variance of team attitude for medical students and nursing students. Blue bars indicate results for Group 1 (received IPE simulation followed by SPE simulation); orange bars indicate results for Group 2 (received SPE simulation followed by IPE simulation). MD, mean difference; IPE, interprofessional education; SPE, single-profession education.
